# Supplementary figures and images for: Competitive binding of MatP and topoisomerase IV to the MukB hinge domain
Source: eLife. 2021 Sep 29;10:e70444. doi: 10.7554/eLife.70444 (PMC8523169; doi:10.7554/eLife.70444)

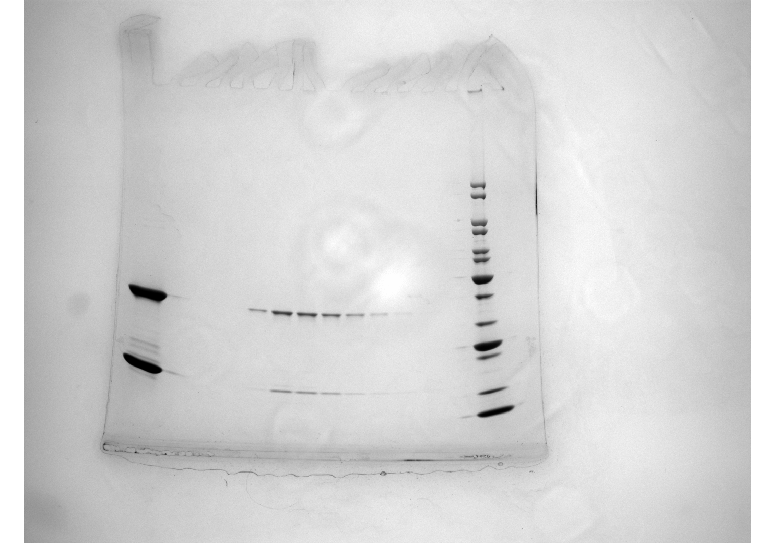

Supplement: Figure 2—source data 1. [file elife-70444-fig2-data1.zip › Figure 2-source data 1/2020_07_01_analytical SEC_LH + MatP.tif]

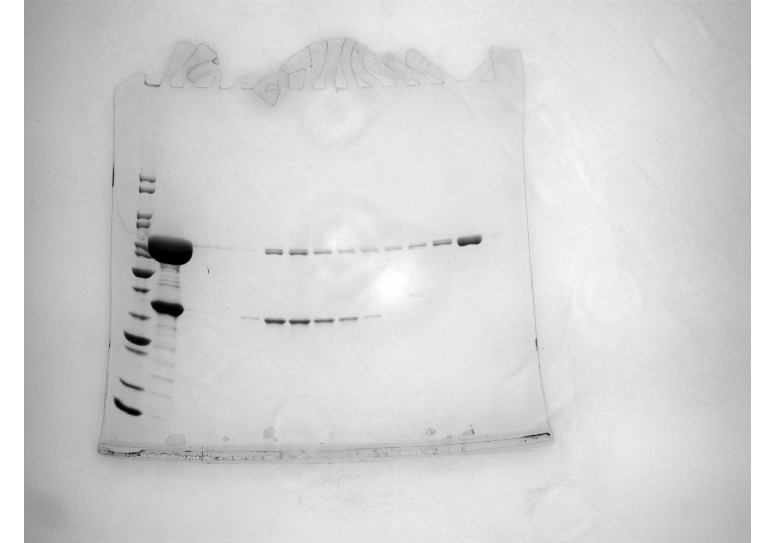

Supplement: Figure 2—source data 1. [file elife-70444-fig2-data1.zip › Figure 2-source data 1/2020_07_01_analytical SEC_LH + ParC.tif]

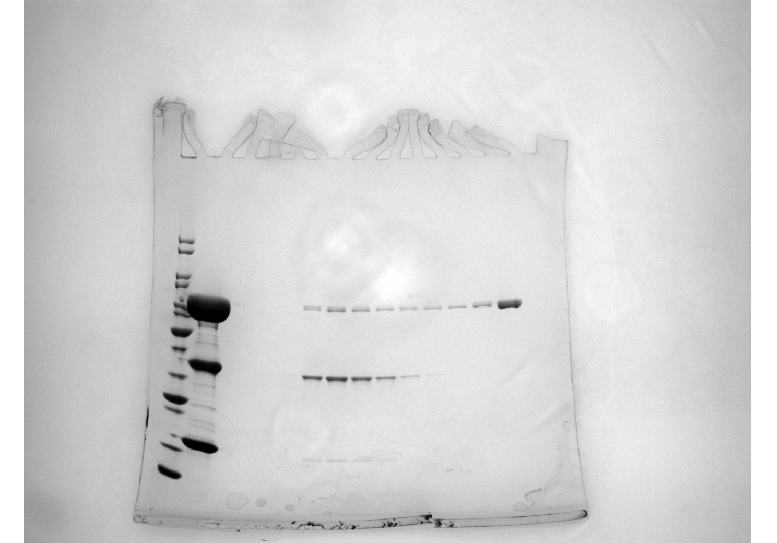

Supplement: Figure 2—source data 1. [file elife-70444-fig2-data1.zip › Figure 2-source data 1/2020_07_01_analytical SEC_LH+ParC+MatP.tif]

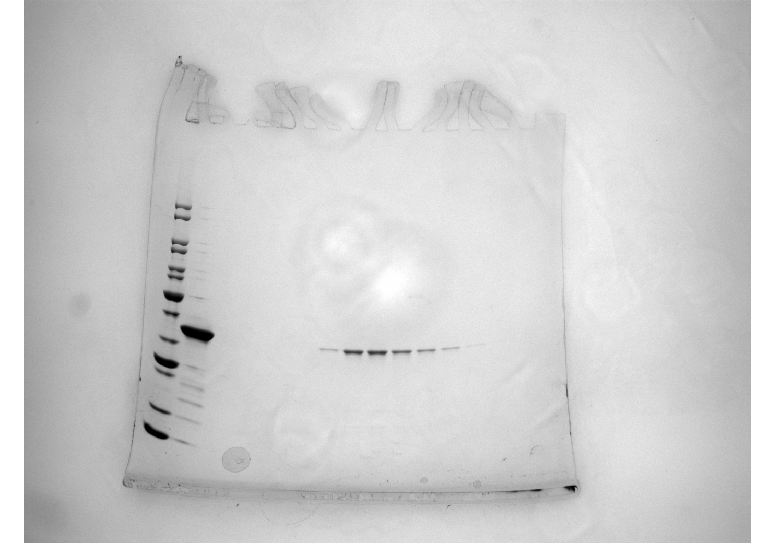

Supplement: Figure 2—source data 1. [file elife-70444-fig2-data1.zip › Figure 2-source data 1/2020_07_01_analytical SEC_LH.tif]

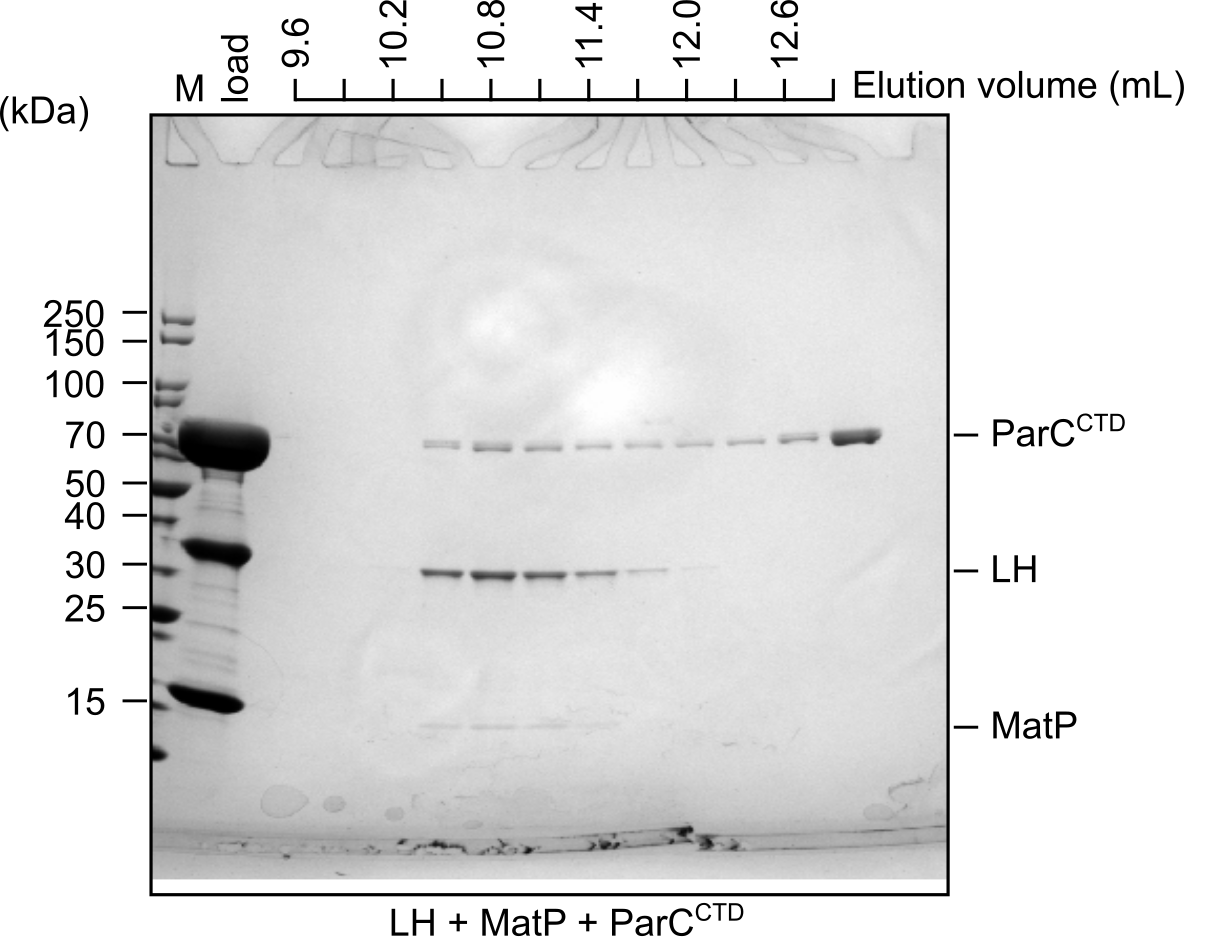

Supplement: Figure 2—source data 1. [file elife-70444-fig2-data1.zip › Figure 2-source data 1/LH + MatP + ParCCTD_uncropped and labelled.png]

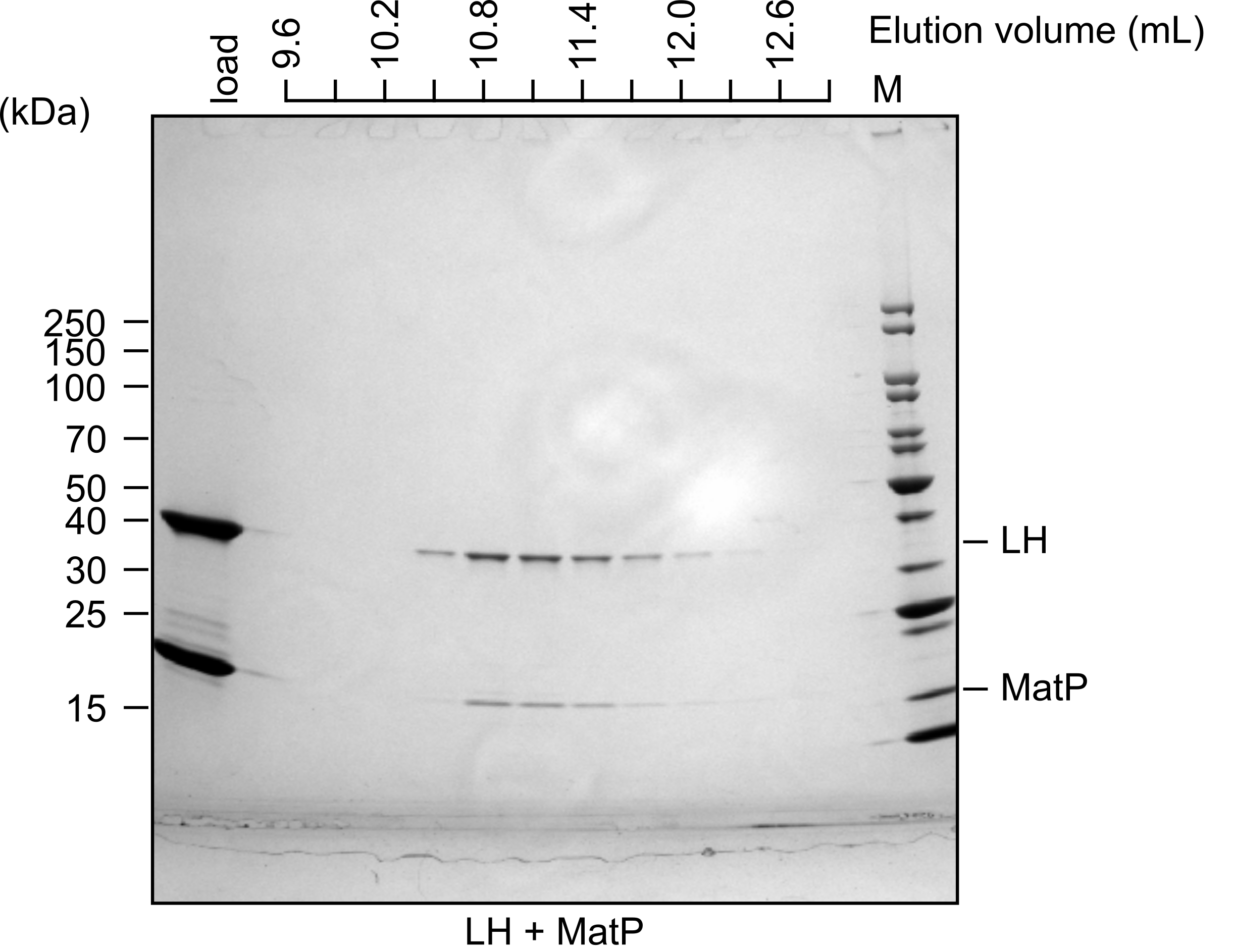

Supplement: Figure 2—source data 1. [file elife-70444-fig2-data1.zip › Figure 2-source data 1/LH + MatP_uncropped and labelled.png]

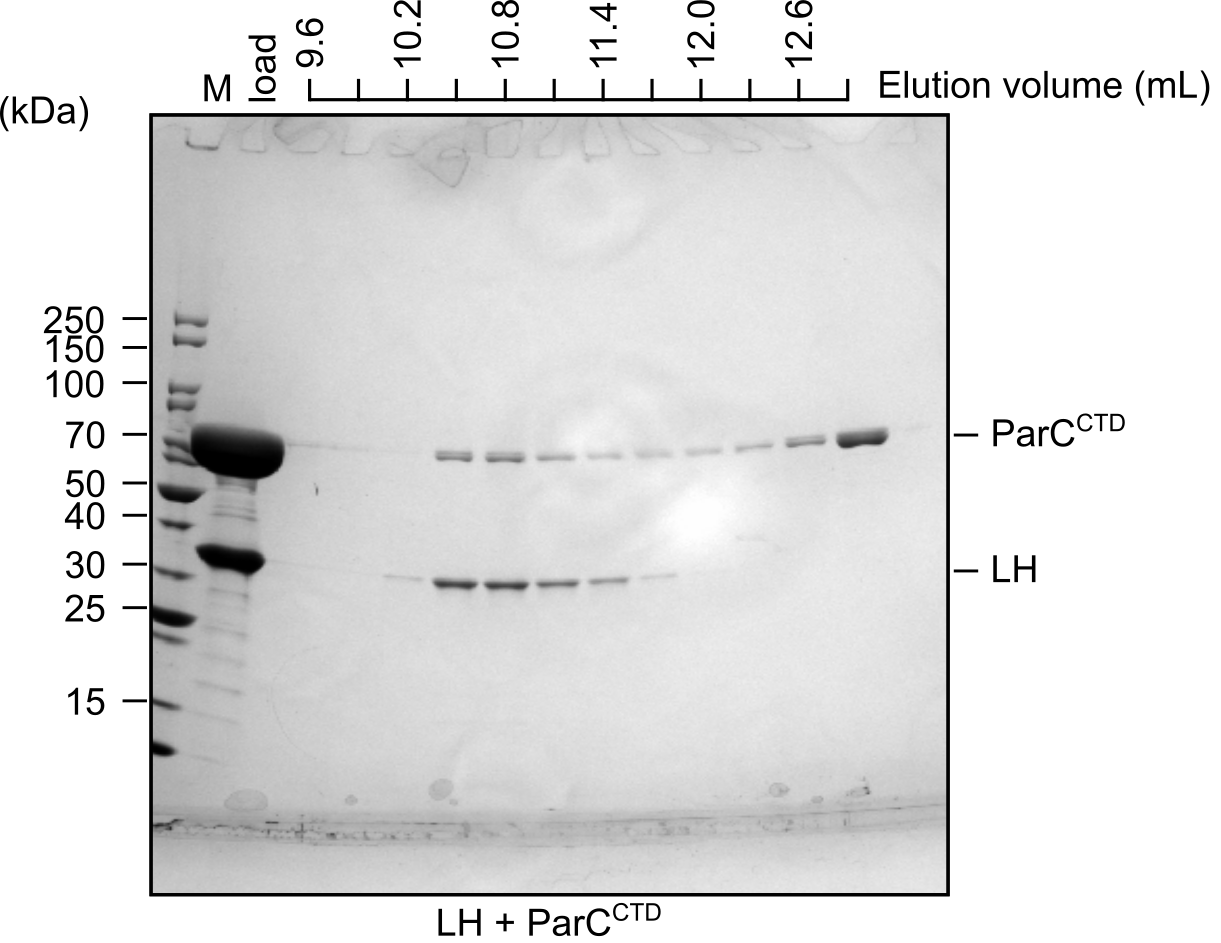

Supplement: Figure 2—source data 1. [file elife-70444-fig2-data1.zip › Figure 2-source data 1/LH + ParCCTD_uncropped and labelled.png]

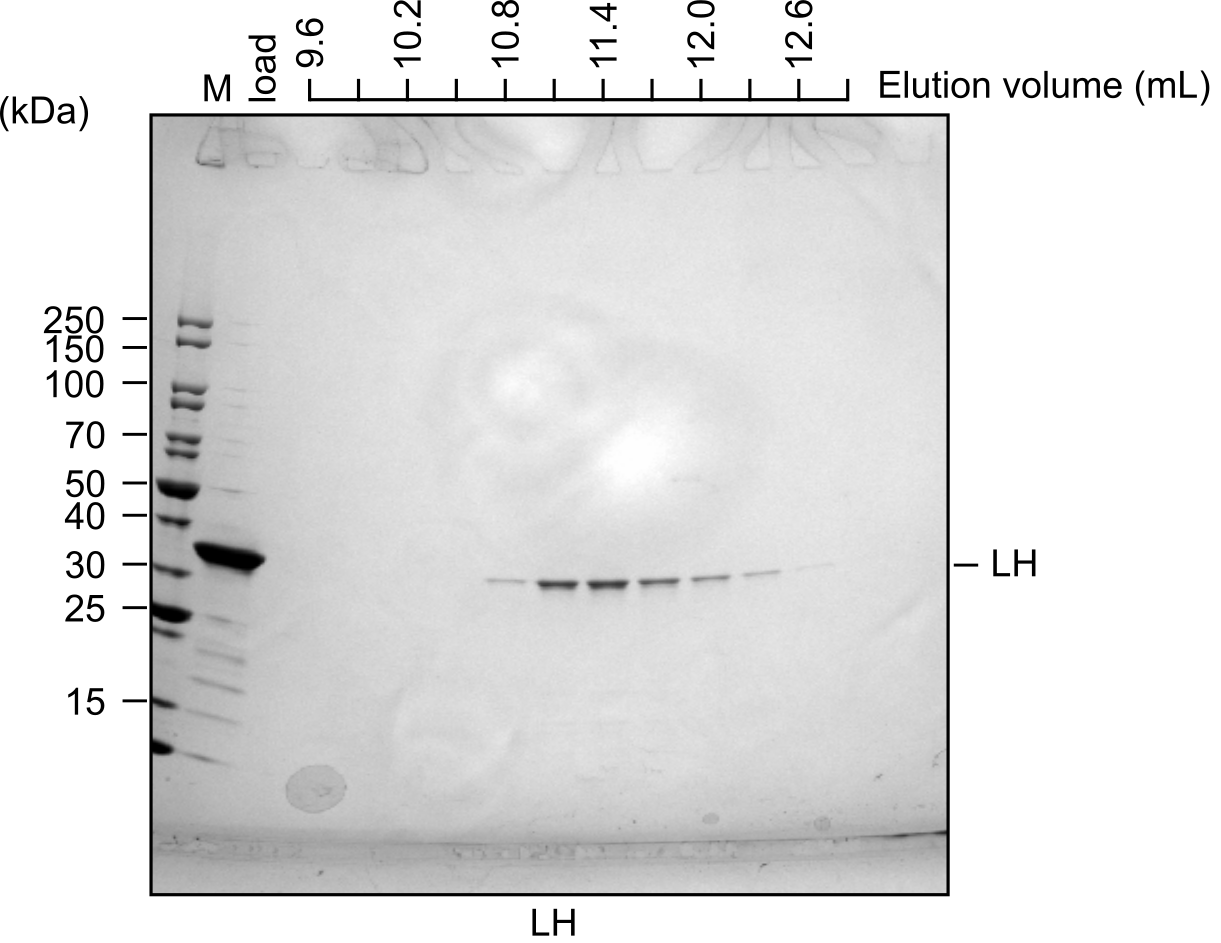

Supplement: Figure 2—source data 1. [file elife-70444-fig2-data1.zip › Figure 2-source data 1/LH alone_uncropped and labelled.png]

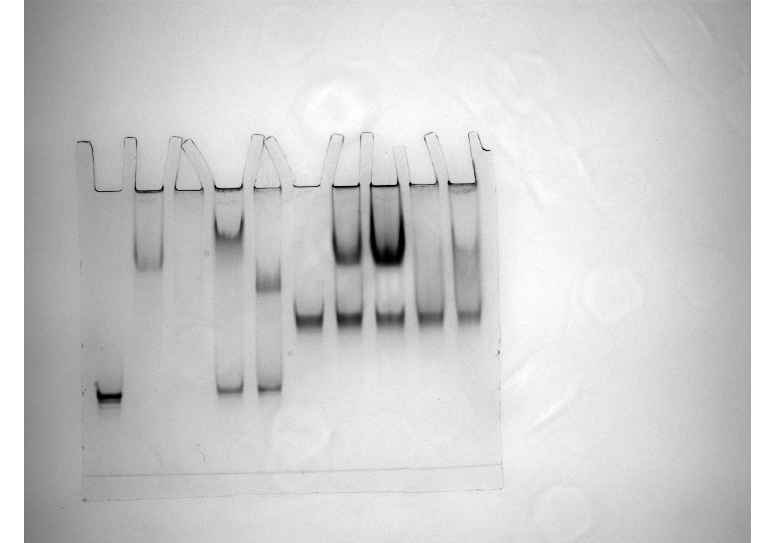

Supplement: Figure 3—source data 1. [file elife-70444-fig3-data1.zip › Figure 3-source data 1/2020_08_07_nativePAGE_GF.tif]

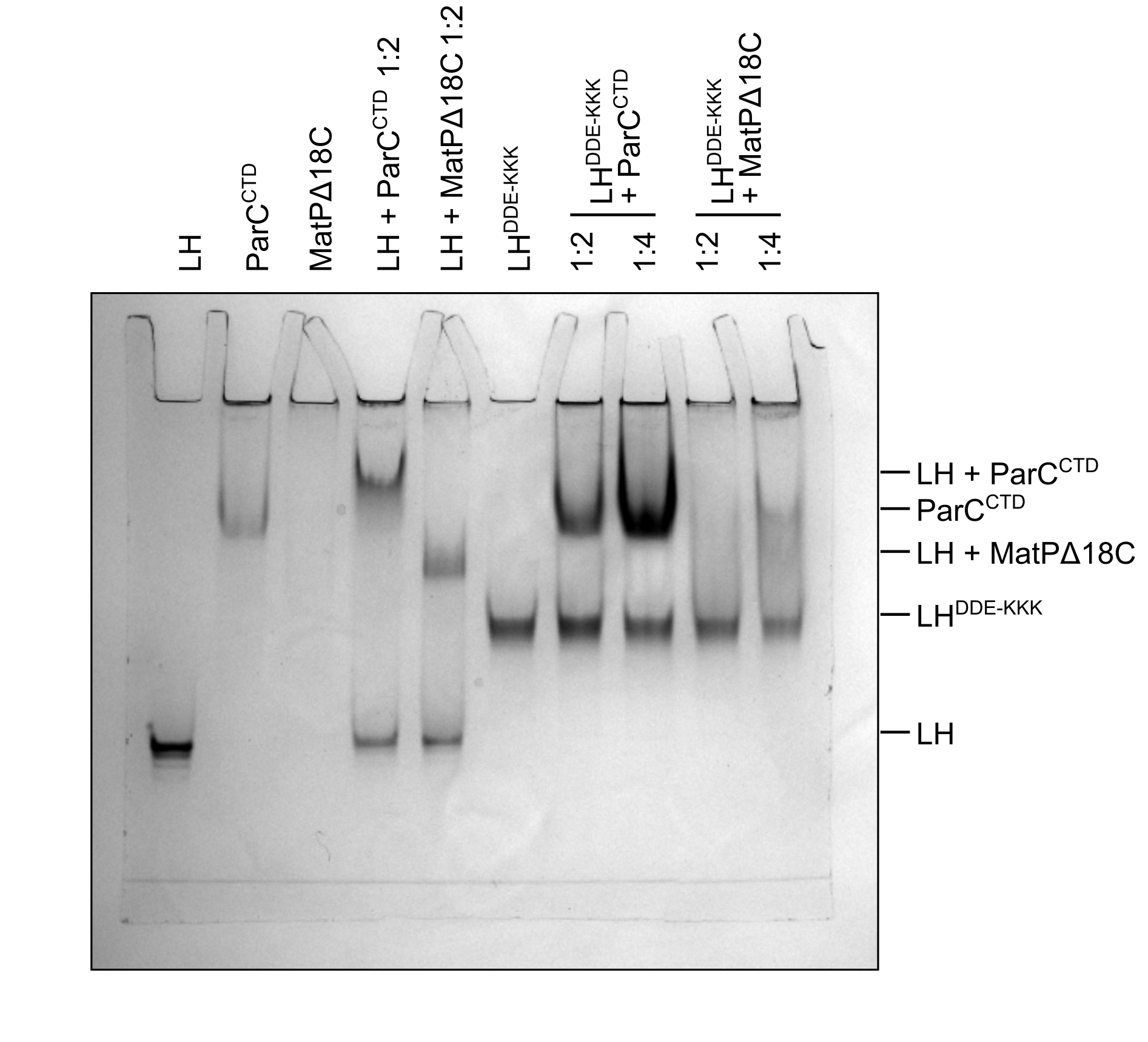

Supplement: Figure 3—source data 1. [file elife-70444-fig3-data1.zip › Figure 3-source data 1/2020_08_11_uncropped and labelled.png]

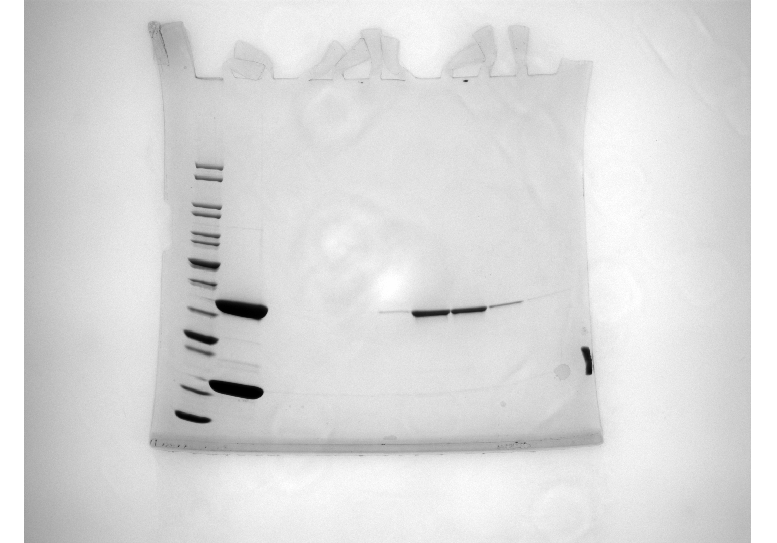

Supplement: Figure 3—source data 2. [file elife-70444-fig3-data2.zip › Figure 3-source data 2/2020_08_11_analytical SEC_LH KKK + MatP.tif]

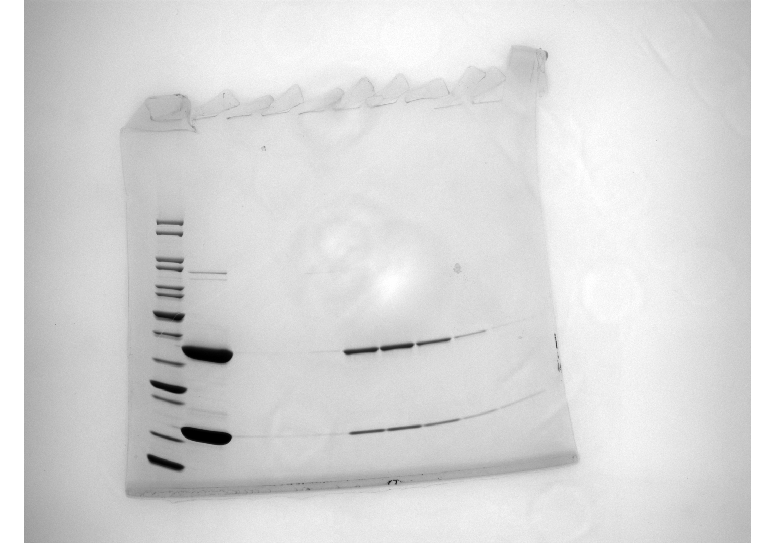

Supplement: Figure 3—source data 2. [file elife-70444-fig3-data2.zip › Figure 3-source data 2/2020_08_11_analytical SEC_LH WT + MatP.tif]

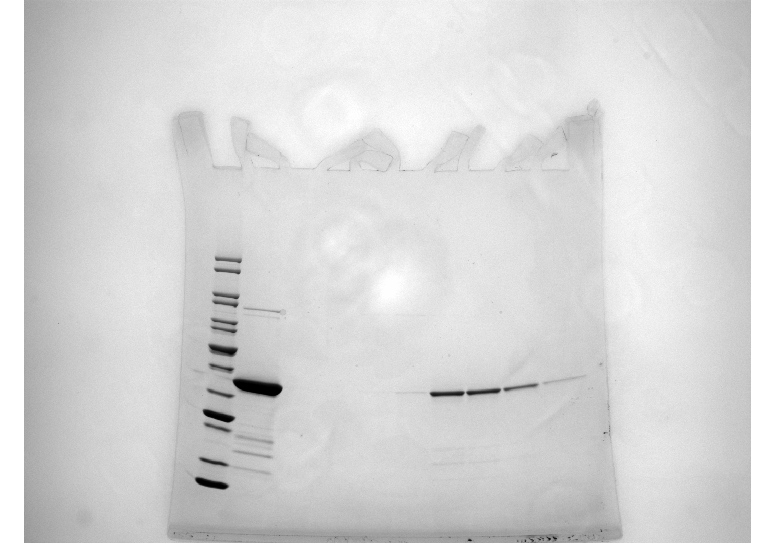

Supplement: Figure 3—source data 2. [file elife-70444-fig3-data2.zip › Figure 3-source data 2/2020_08_11_analytical SEC_LH WT.tif]

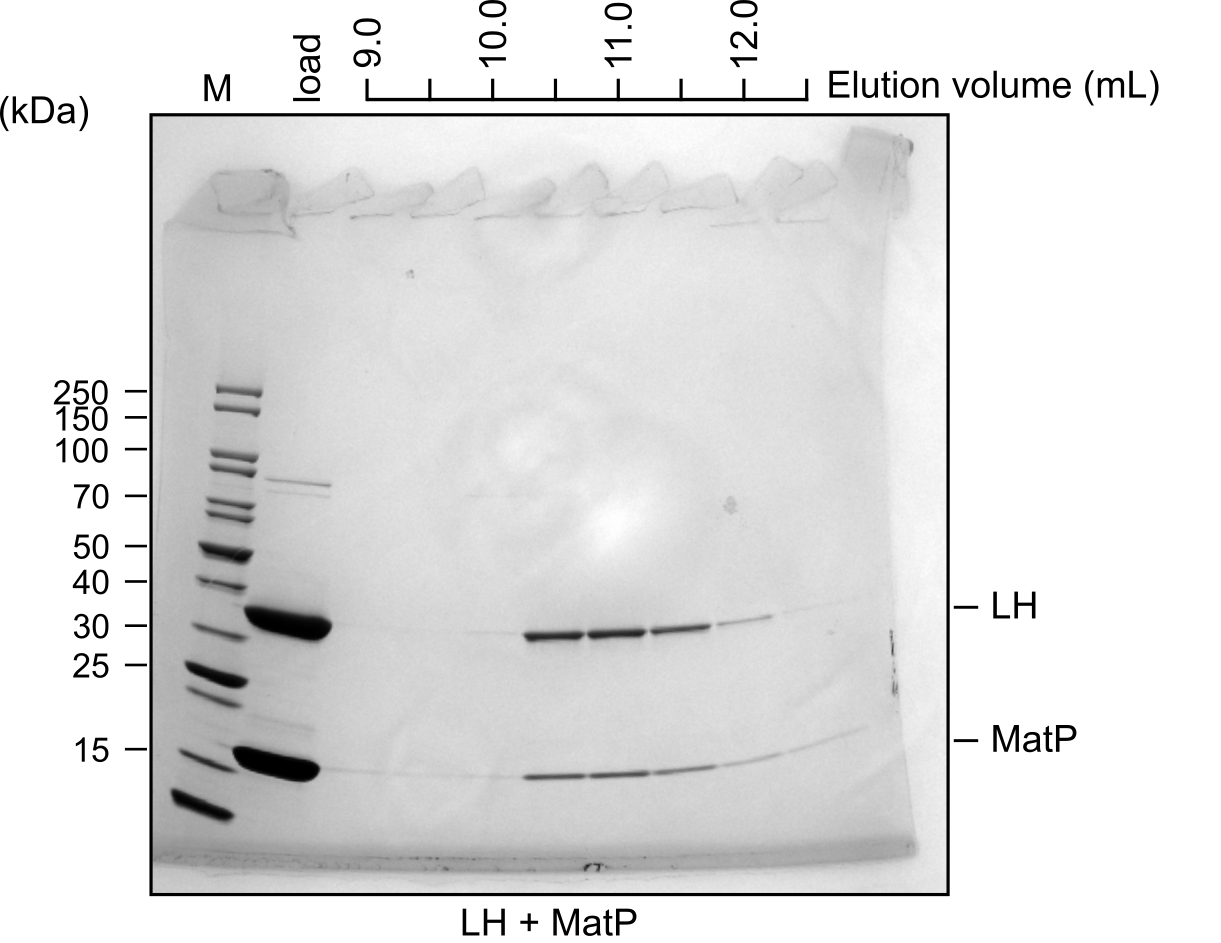

Supplement: Figure 3—source data 2. [file elife-70444-fig3-data2.zip › Figure 3-source data 2/LH + MatP_uncropped and labelled.png]

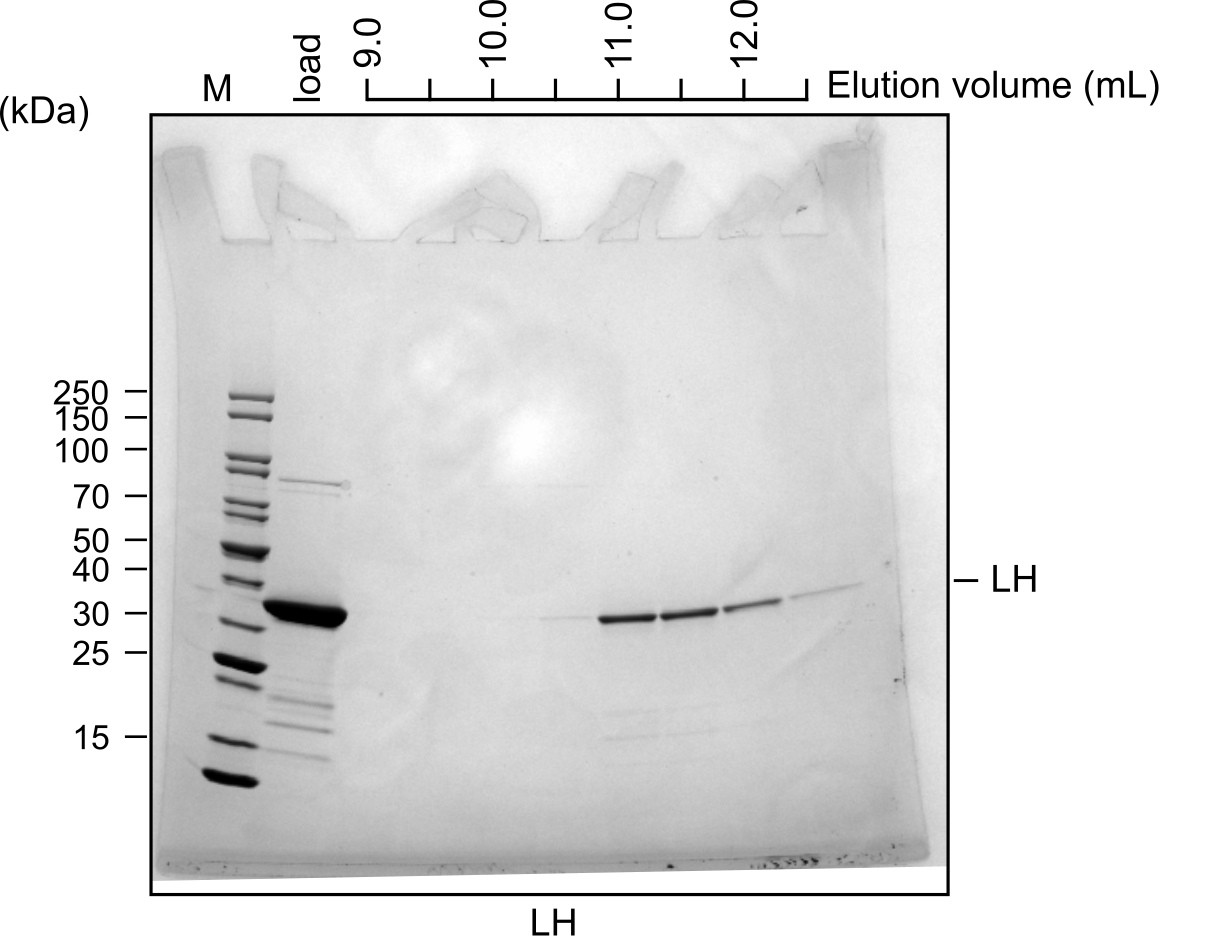

Supplement: Figure 3—source data 2. [file elife-70444-fig3-data2.zip › Figure 3-source data 2/LH alone_uncropped and labelled.png]

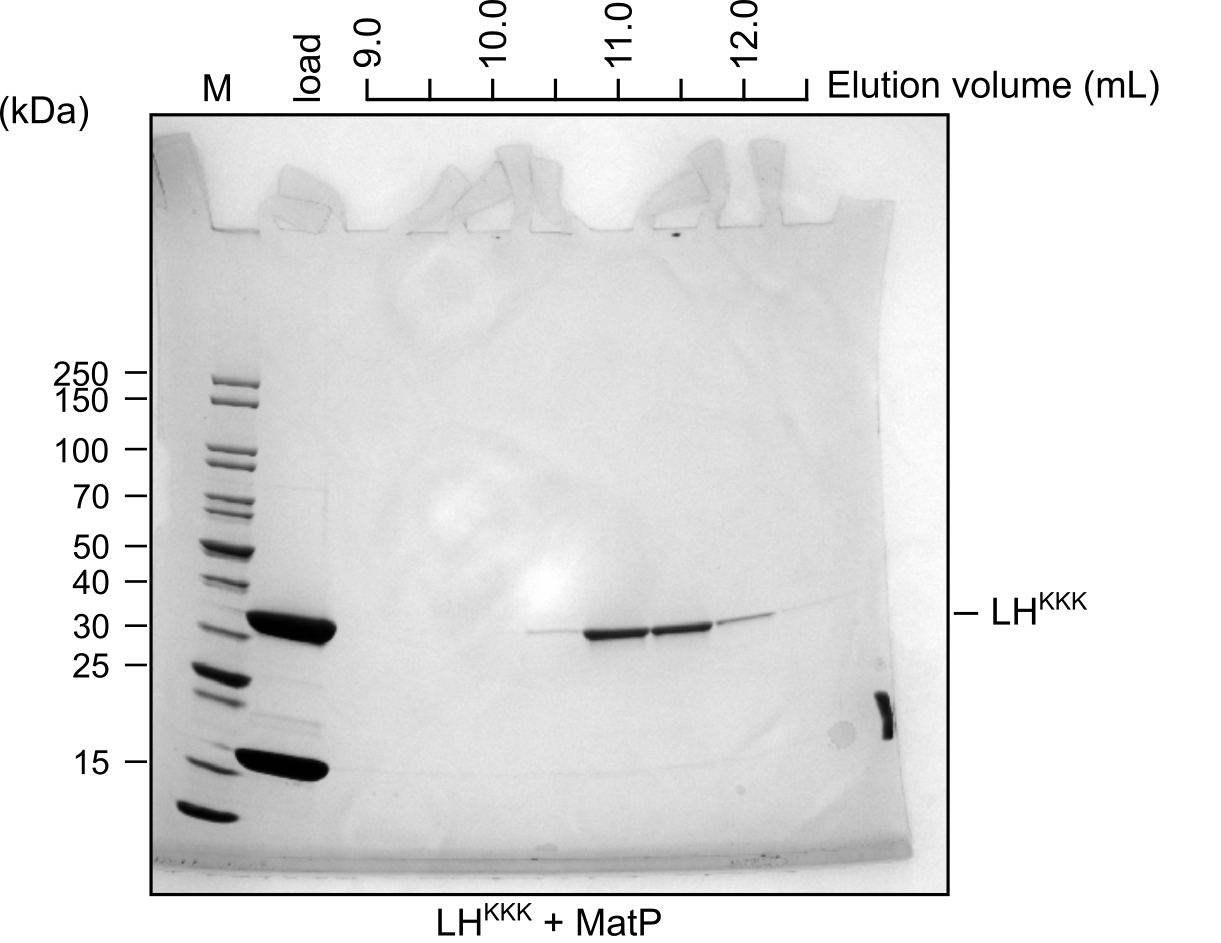

Supplement: Figure 3—source data 2. [file elife-70444-fig3-data2.zip › Figure 3-source data 2/LH KKK+MatP_uncropped and labelled.png]

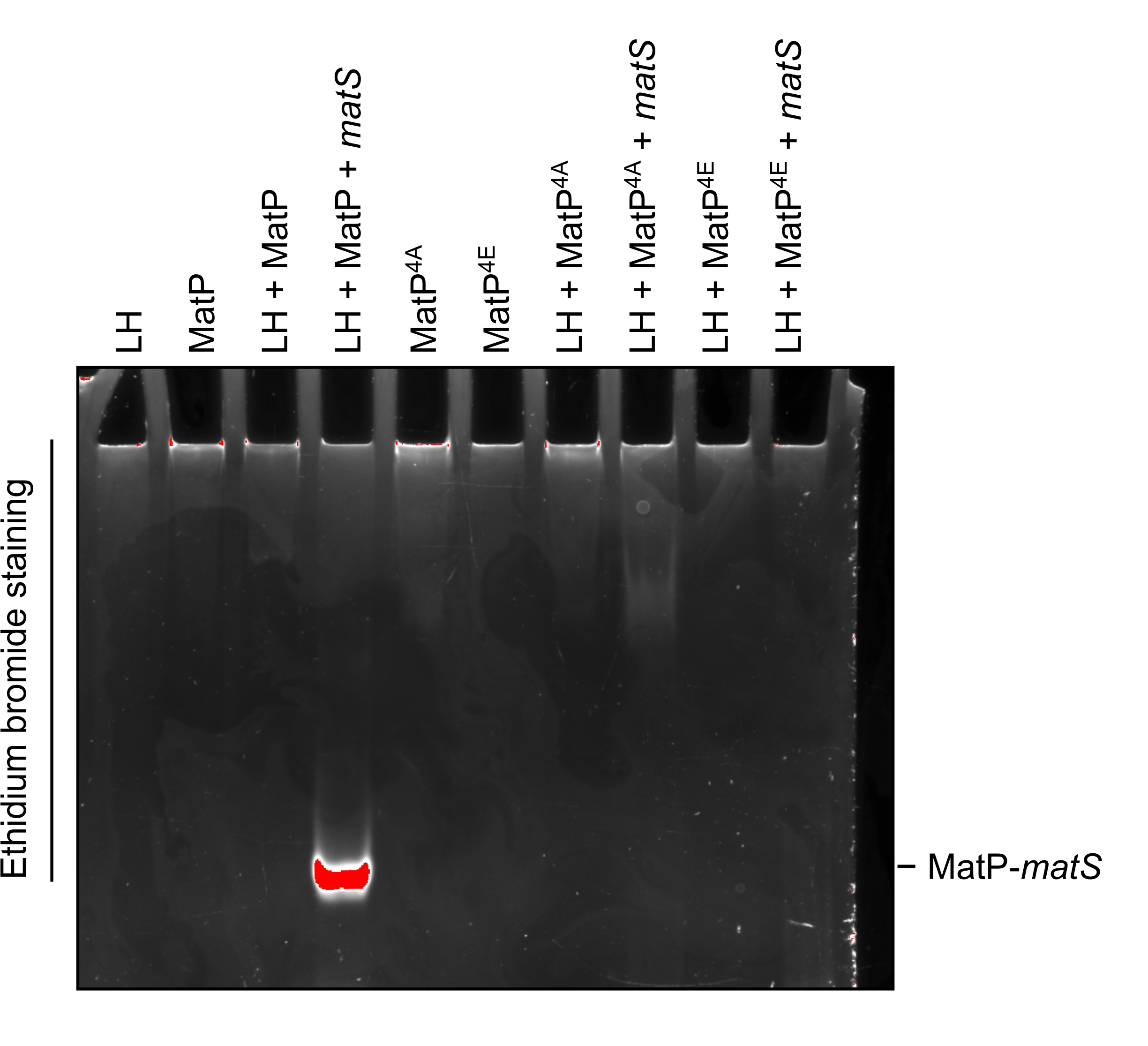

Supplement: Figure 4—source data 1. [file elife-70444-fig4-data1.zip › Figure 4-source data 1/171120_MatP-matS_ethidium_uncropped and labelled.png]

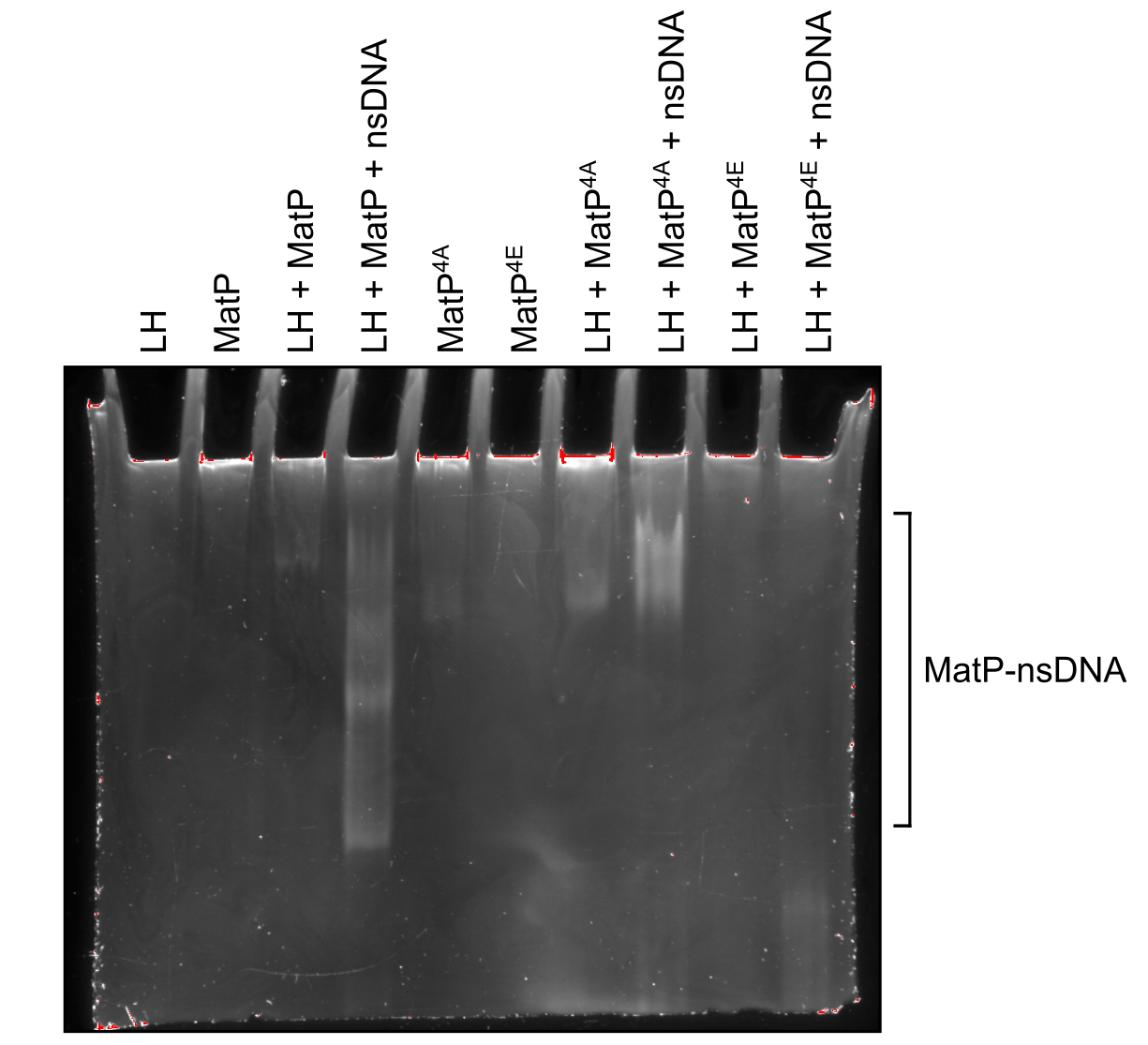

Supplement: Figure 4—source data 1. [file elife-70444-fig4-data1.zip › Figure 4-source data 1/171120_MatP-nsDNA_ethidium_uncropped and labelled.png]

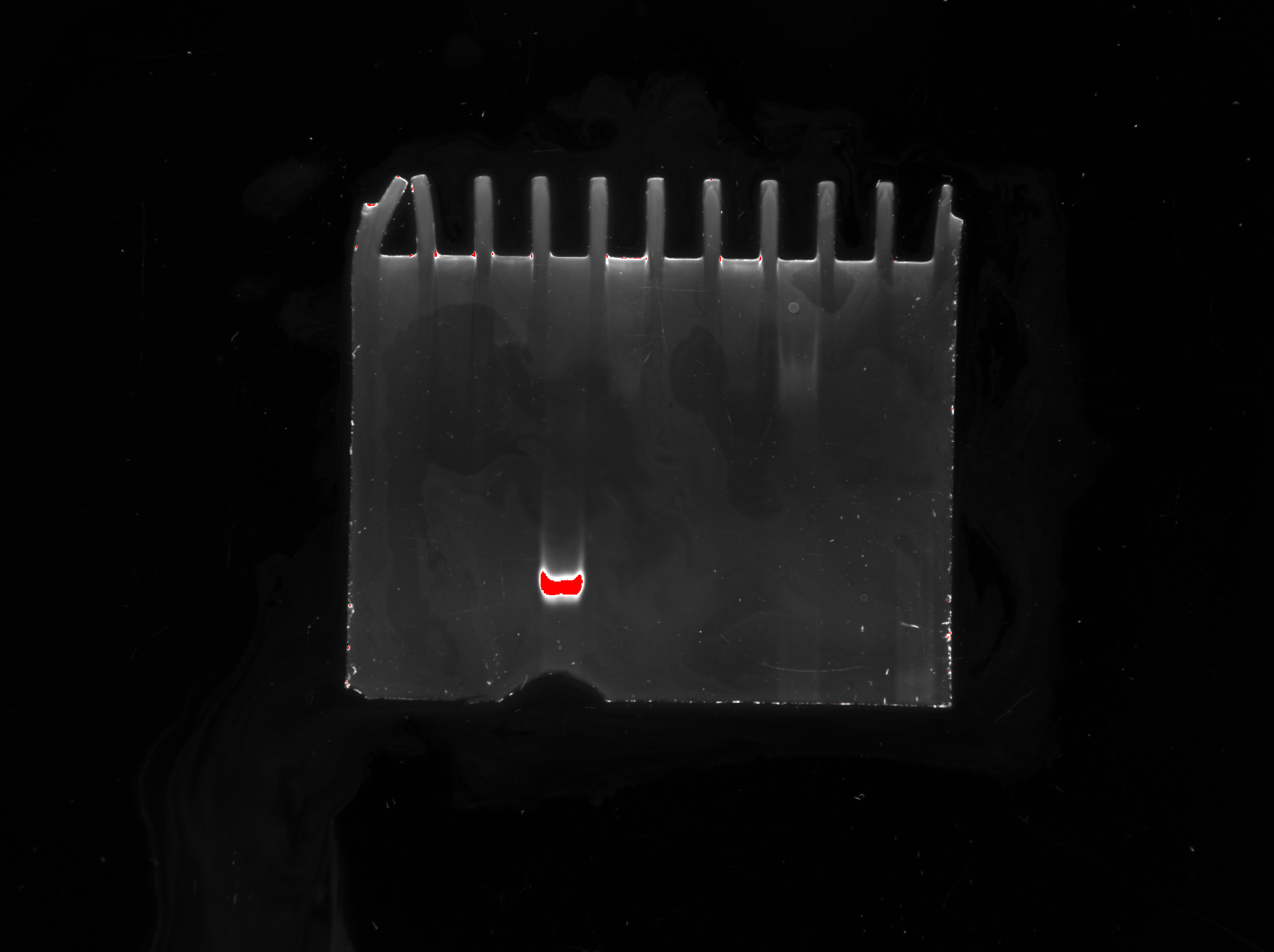

Supplement: Figure 4—source data 1. [file elife-70444-fig4-data1.zip › Figure 4-source data 1/171120_MatPmats.tif]

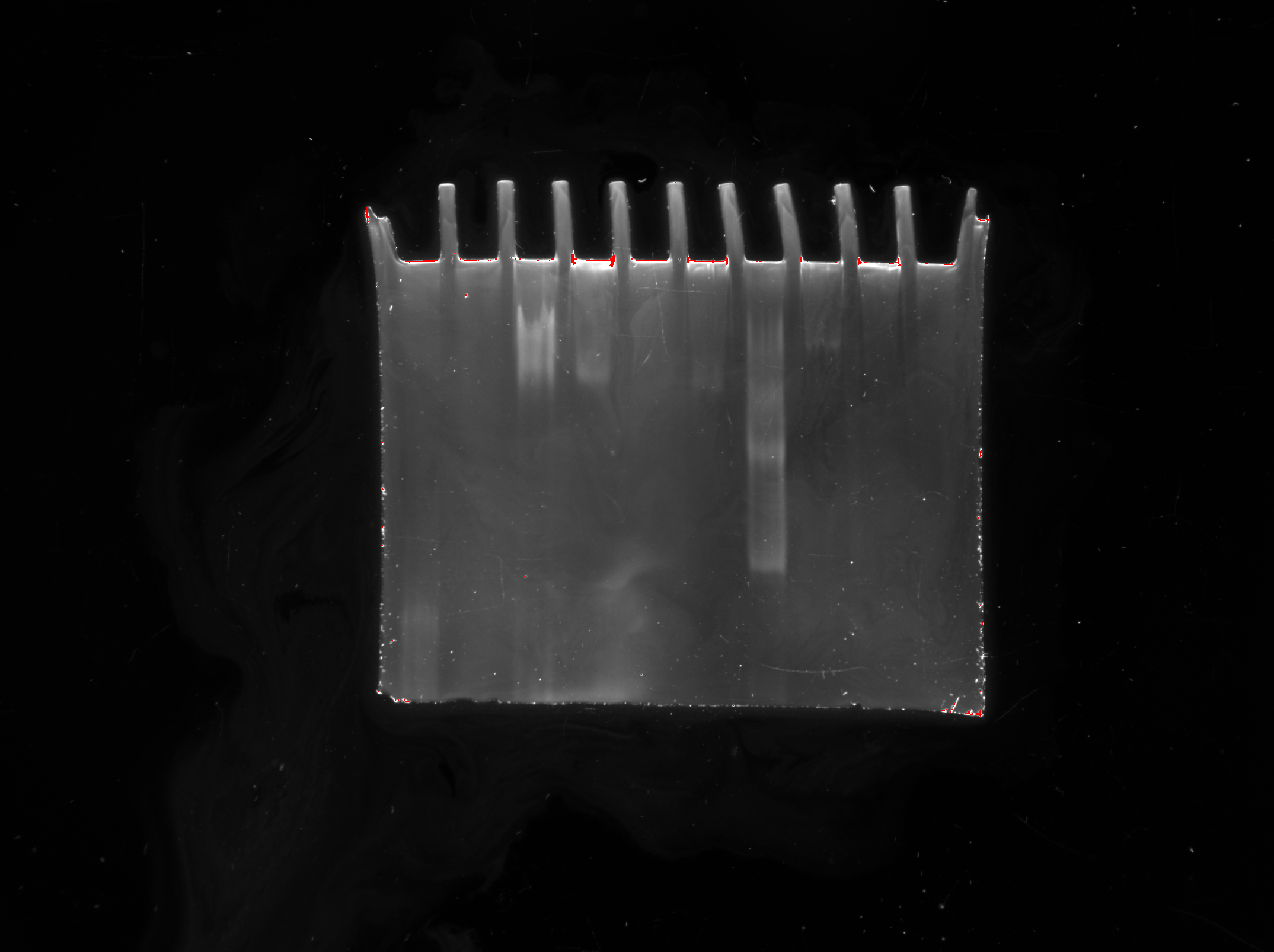

Supplement: Figure 4—source data 1. [file elife-70444-fig4-data1.zip › Figure 4-source data 1/171120_MatPnsDNA.tif]

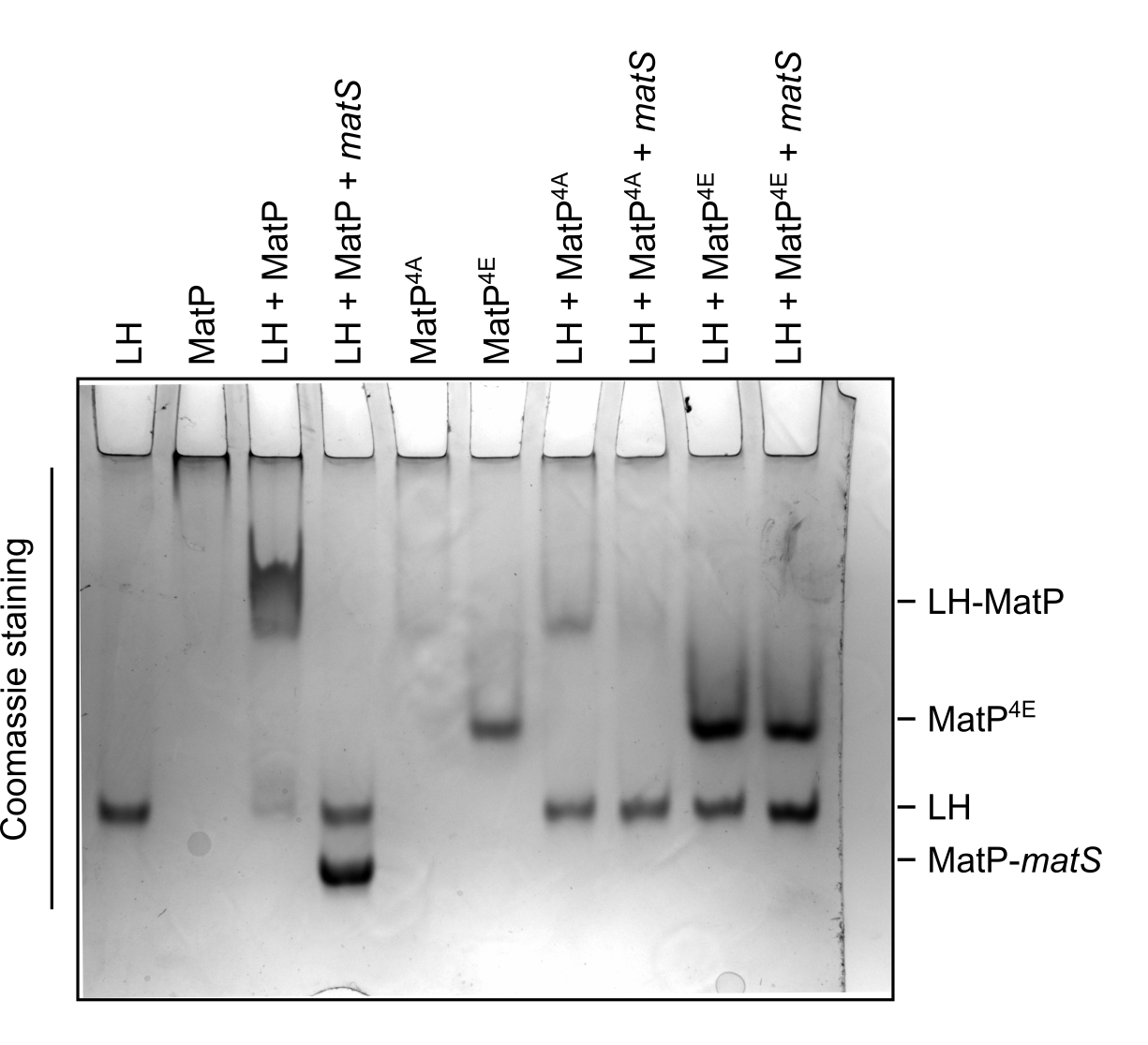

Supplement: Figure 4—source data 1. [file elife-70444-fig4-data1.zip › Figure 4-source data 1/181120_MatP-matS_uncropped and labelled.png]

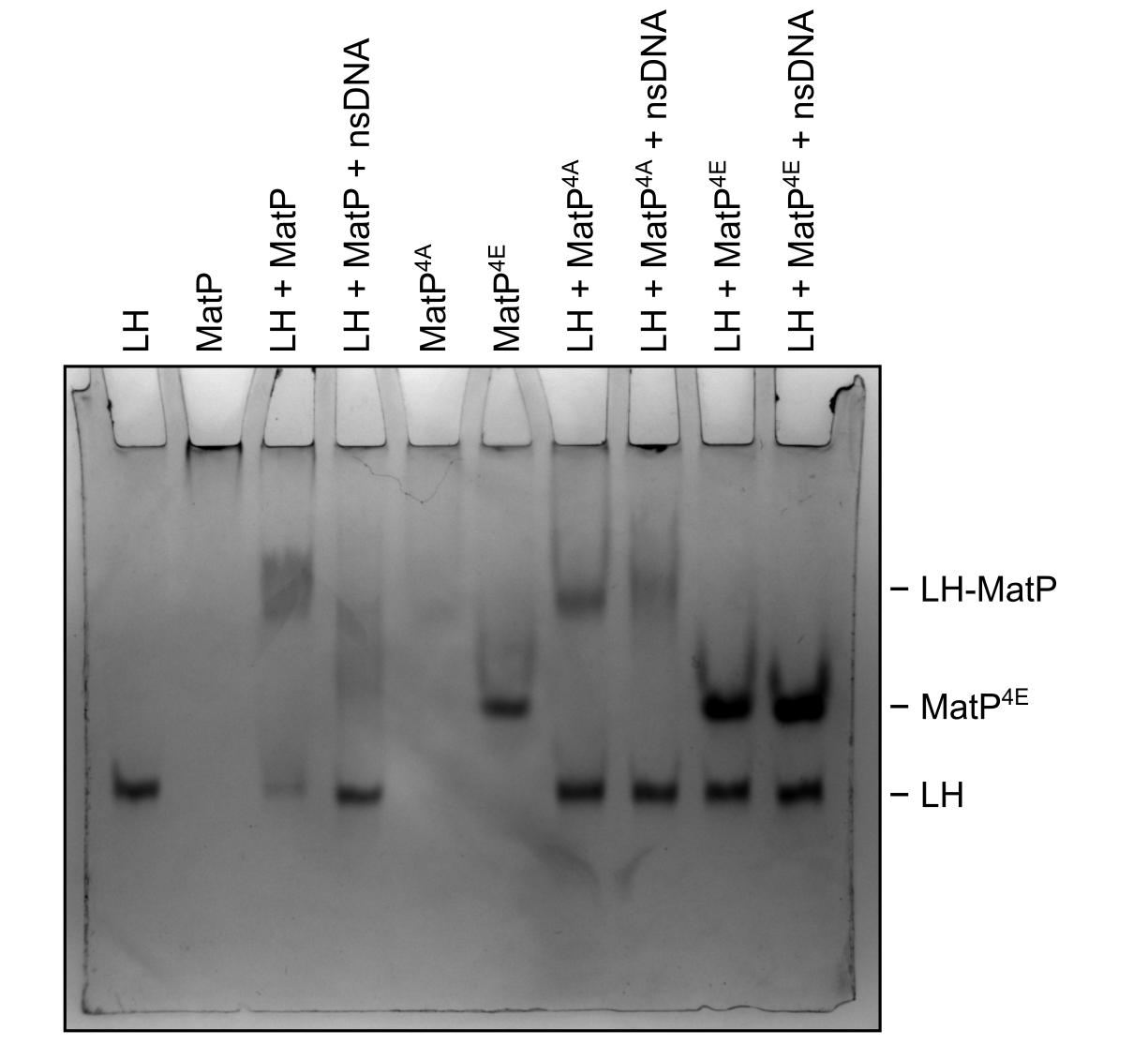

Supplement: Figure 4—source data 1. [file elife-70444-fig4-data1.zip › Figure 4-source data 1/181120_MatP-nsDNA_uncropped and labelled.png]

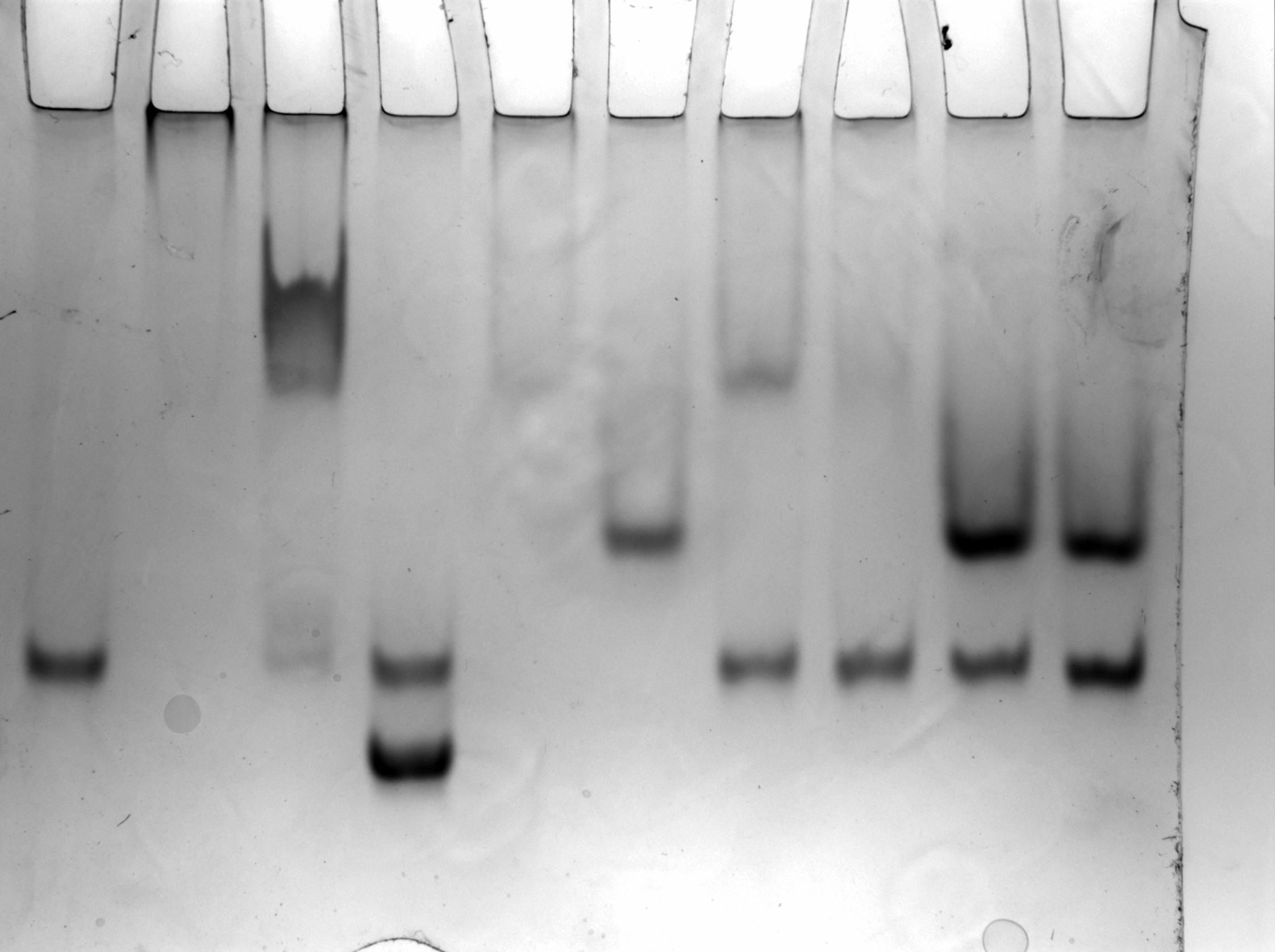

Supplement: Figure 4—source data 1. [file elife-70444-fig4-data1.zip › Figure 4-source data 1/181120_MatPmats.tif]

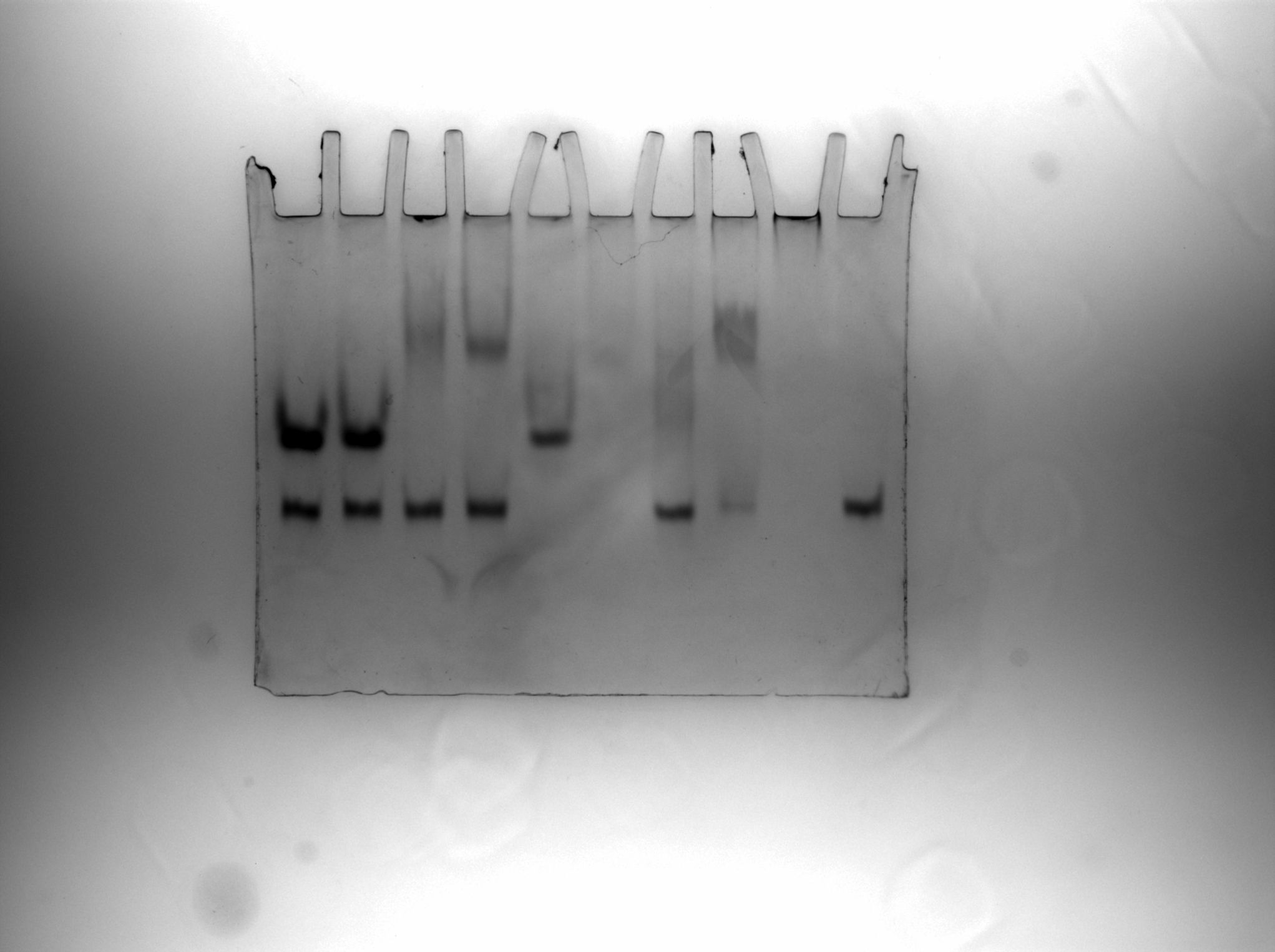

Supplement: Figure 4—source data 1. [file elife-70444-fig4-data1.zip › Figure 4-source data 1/181120_MatPnsDNA.tif]

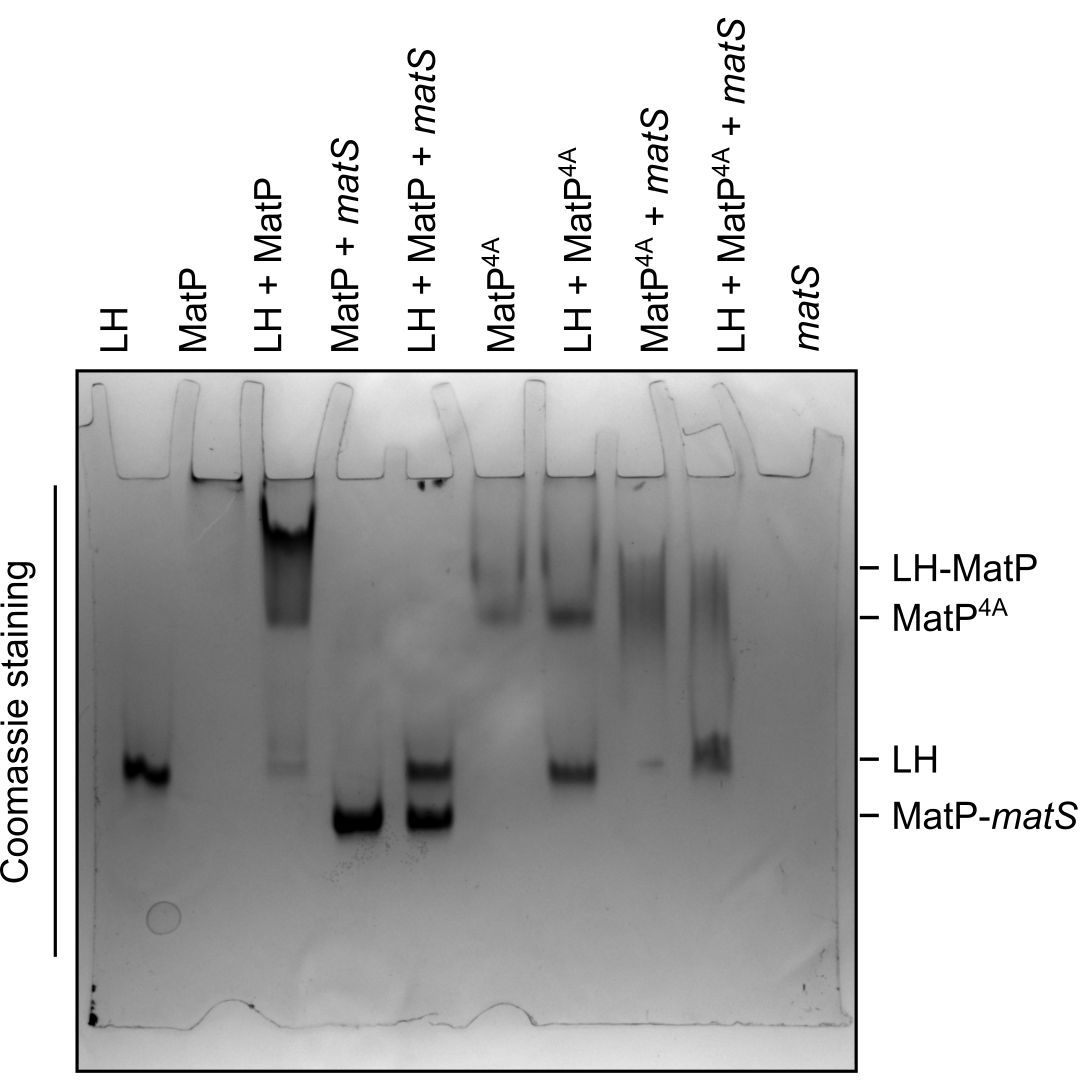

Supplement: Figure 4—figure supplement 1—source data 1. [file elife-70444-fig4-figsupp1-data1.zip › Figure 4-supplement 1-source data 1/111120_MatP4Amutant_matSandLHbinding_Coomassie.png]

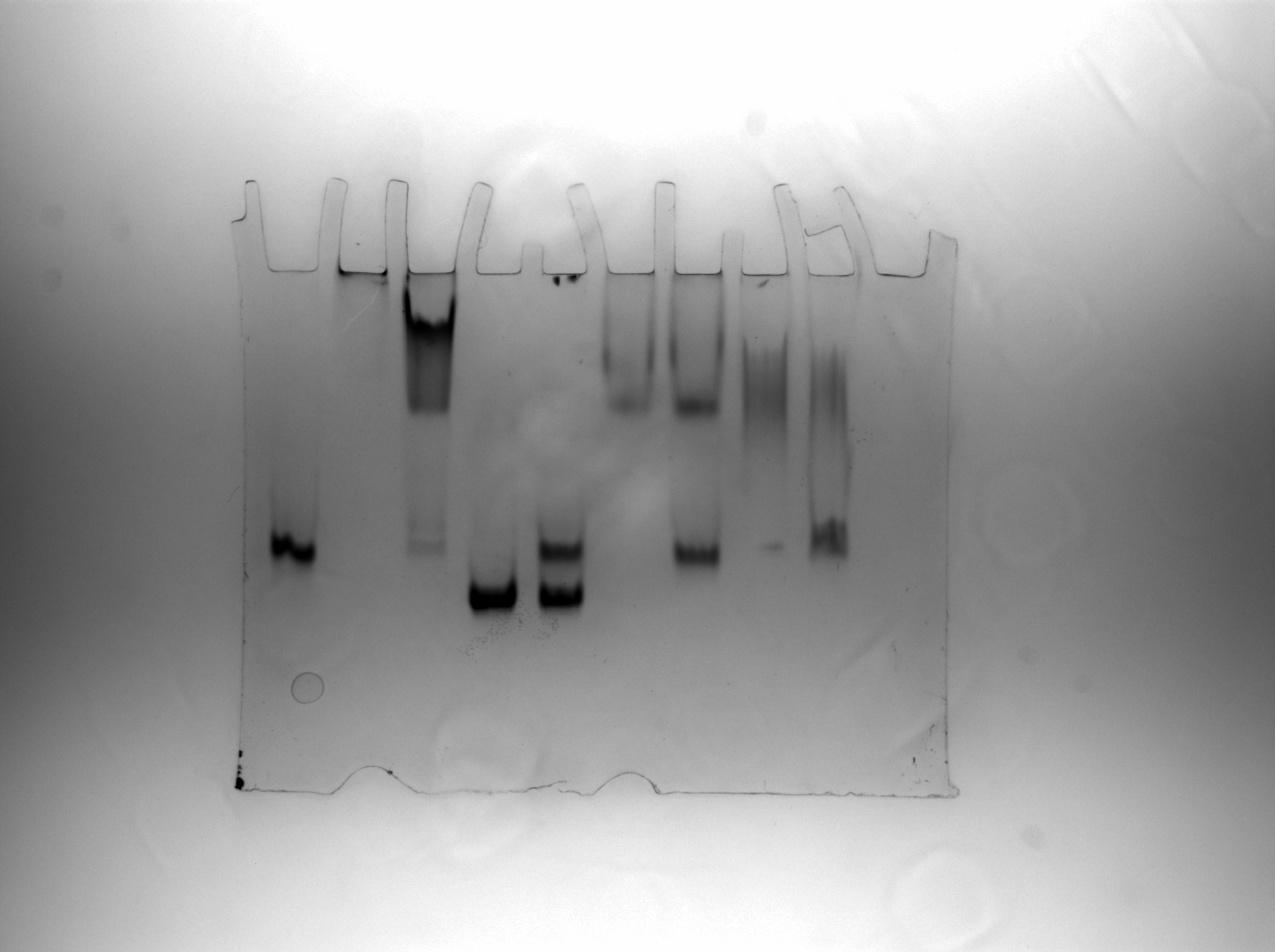

Supplement: Figure 4—figure supplement 1—source data 1. [file elife-70444-fig4-figsupp1-data1.zip › Figure 4-supplement 1-source data 1/111120_MatP4Amutant_matSandLHbinding_Coomassie.tif]

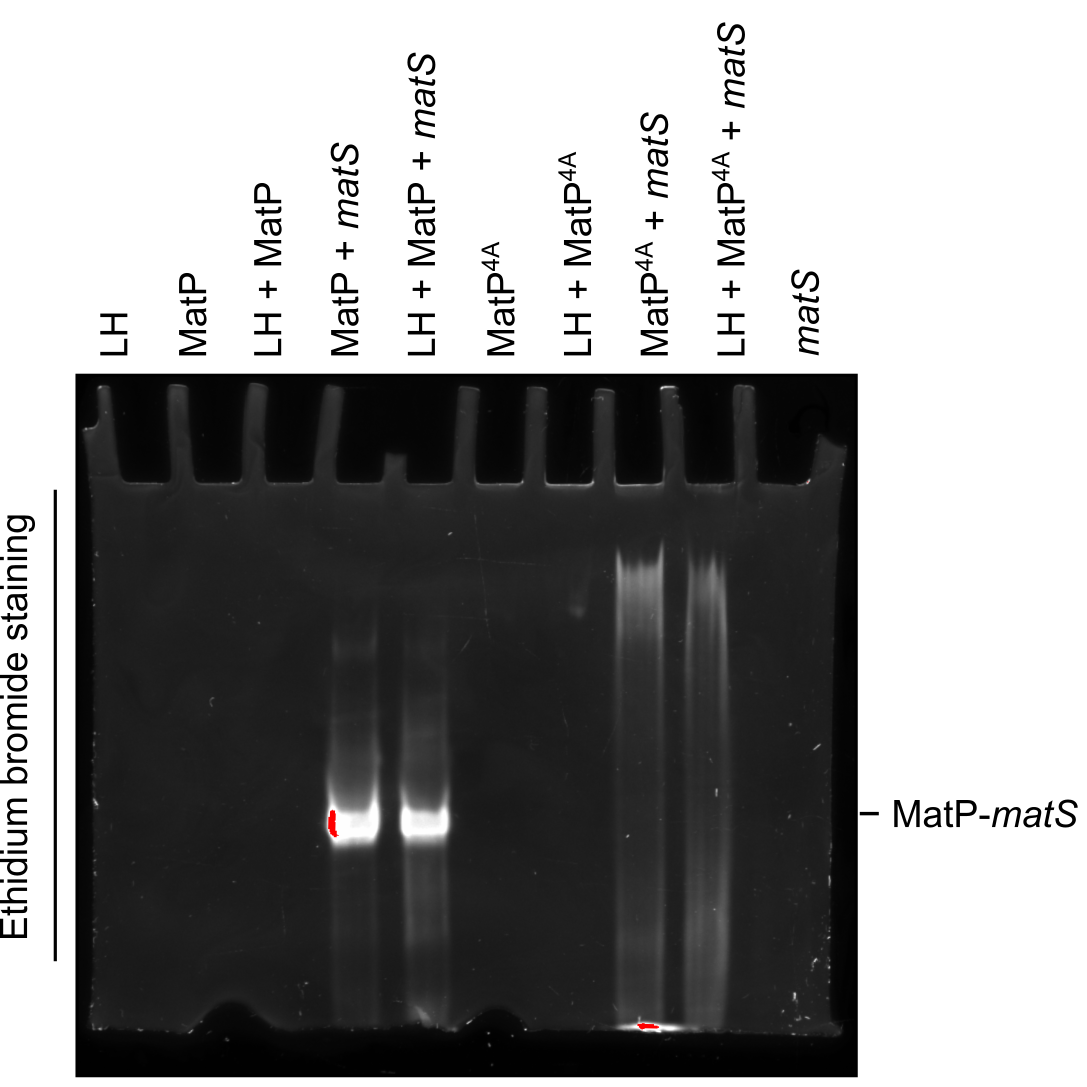

Supplement: Figure 4—figure supplement 1—source data 1. [file elife-70444-fig4-figsupp1-data1.zip › Figure 4-supplement 1-source data 1/111120_MatP4Amutant_matSandLHbinding_ethidium.png]

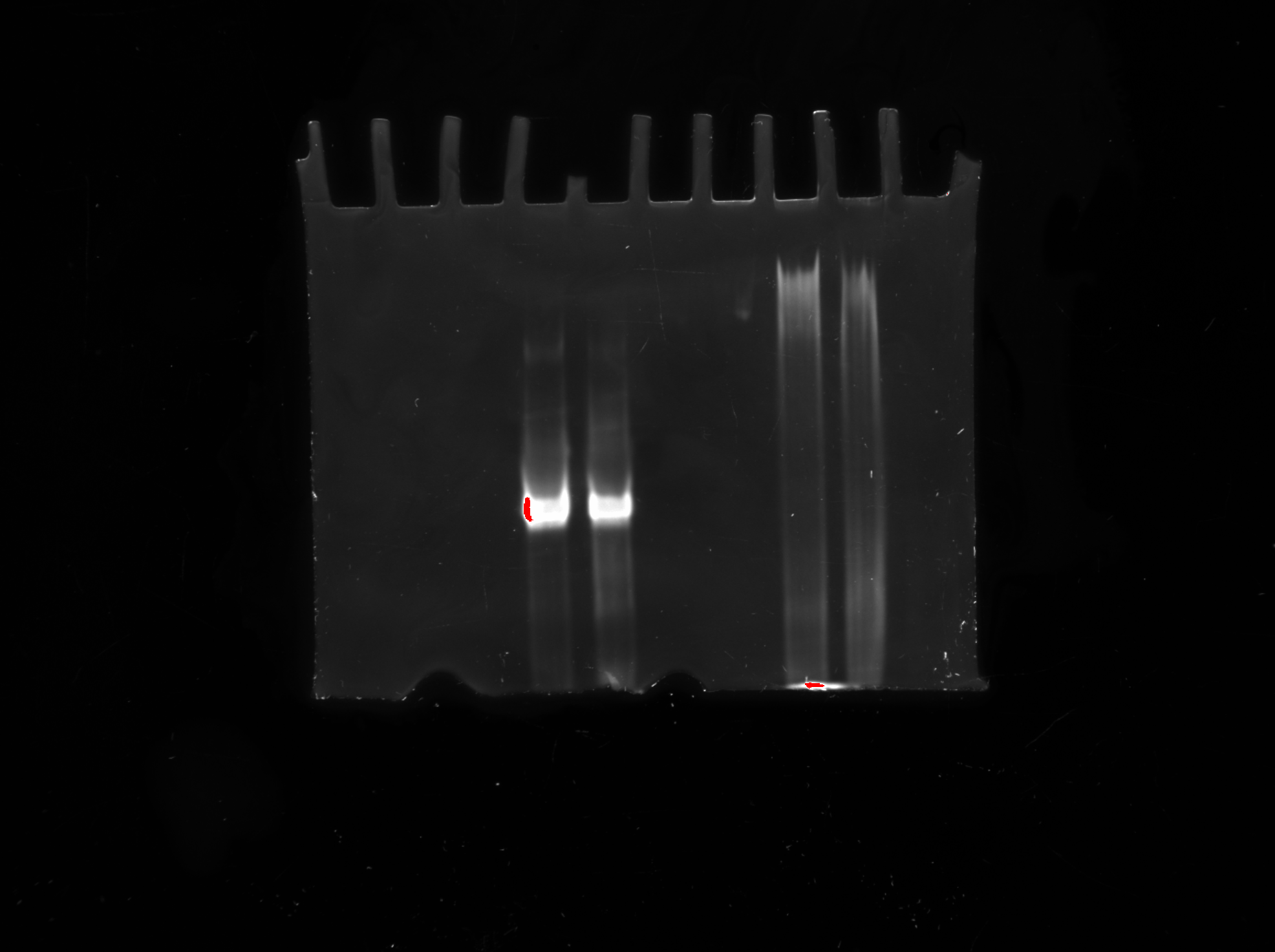

Supplement: Figure 4—figure supplement 1—source data 1. [file elife-70444-fig4-figsupp1-data1.zip › Figure 4-supplement 1-source data 1/111120_MatP4Amutant_matSandLHbinding_ethidium.tif]

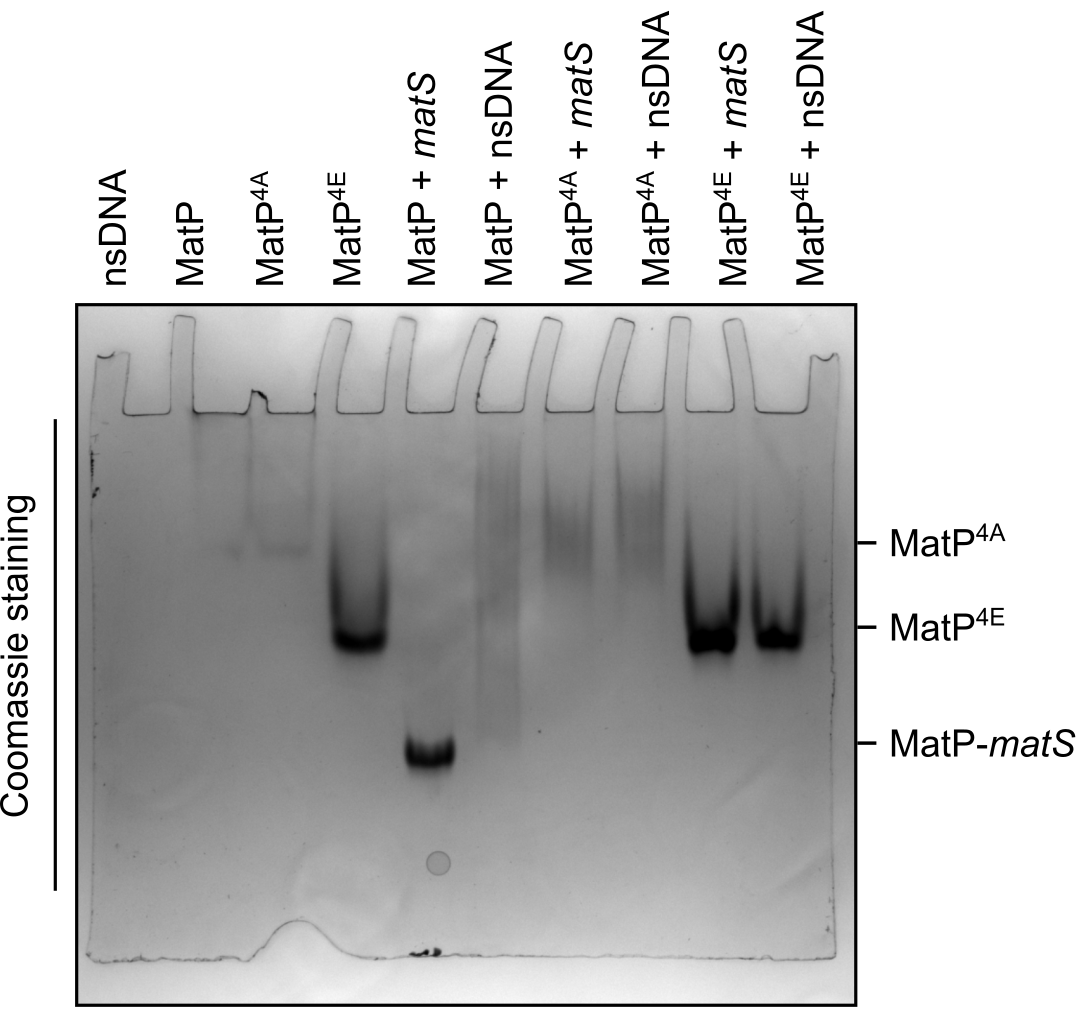

Supplement: Figure 4—figure supplement 1—source data 1. [file elife-70444-fig4-figsupp1-data1.zip › Figure 4-supplement 1-source data 1/111120_MatPmutants_matSvnsDNA_Coomassie.png]

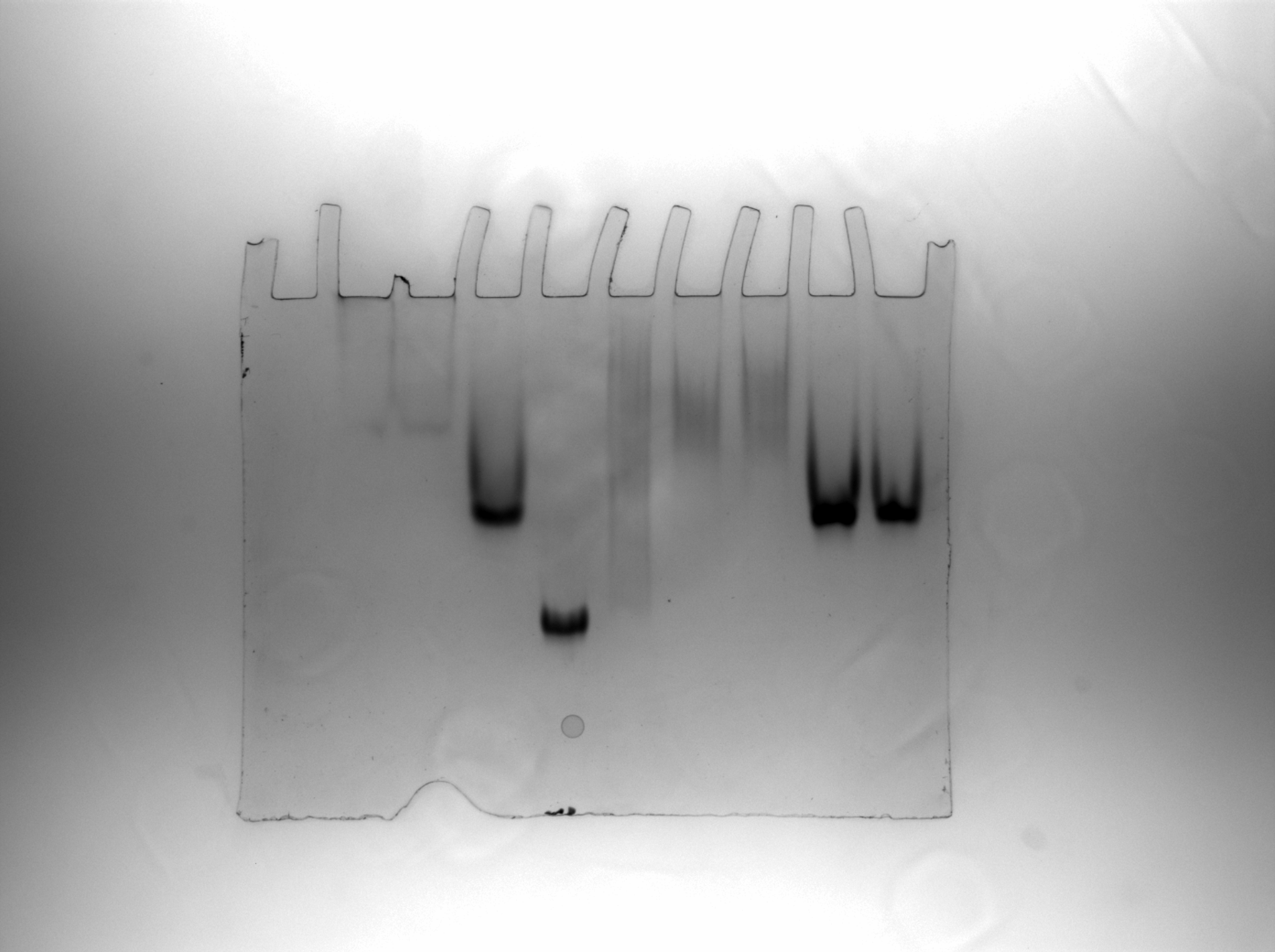

Supplement: Figure 4—figure supplement 1—source data 1. [file elife-70444-fig4-figsupp1-data1.zip › Figure 4-supplement 1-source data 1/111120_MatPmutants_matSvnsDNA_Coomassie.tif]

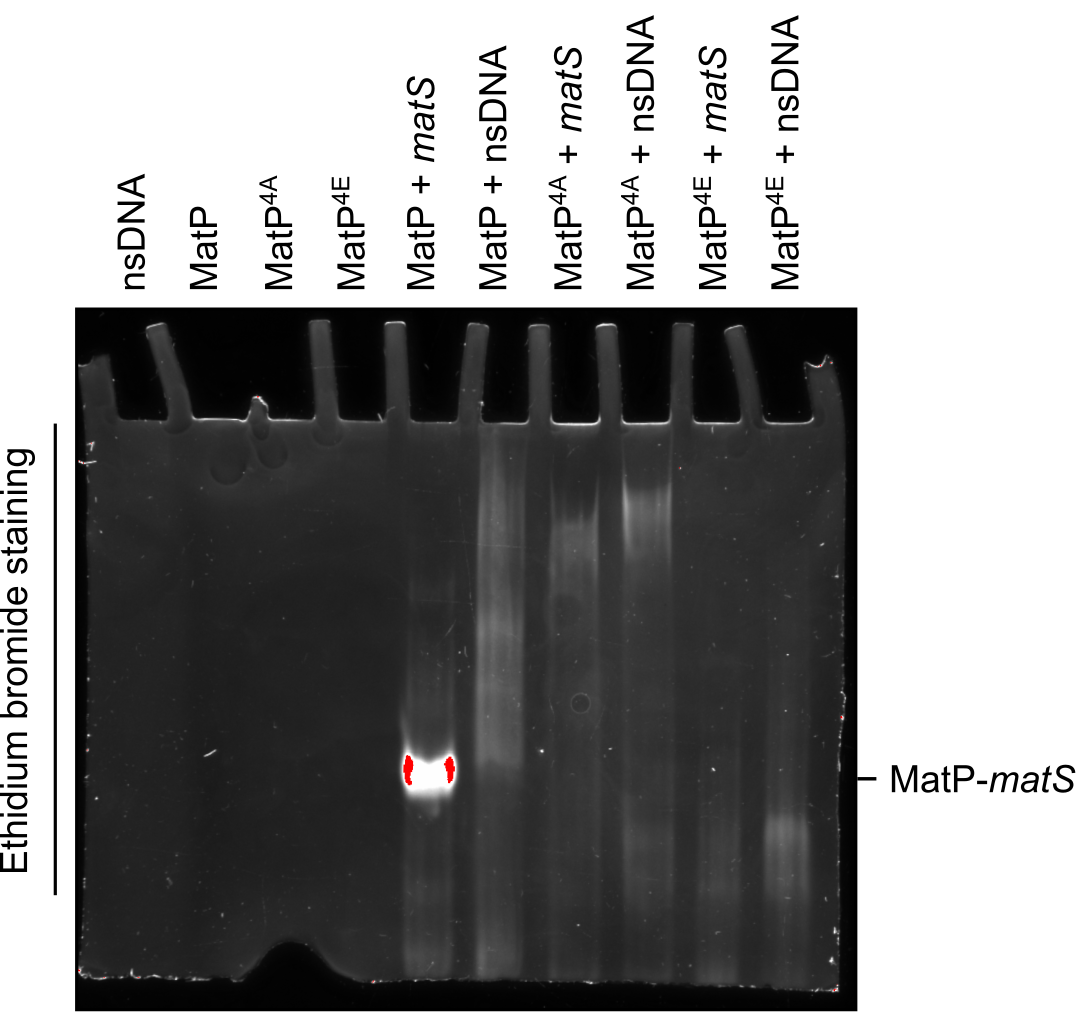

Supplement: Figure 4—figure supplement 1—source data 1. [file elife-70444-fig4-figsupp1-data1.zip › Figure 4-supplement 1-source data 1/111120_MatPmutants_matSvnsDNA_ethidium.png]

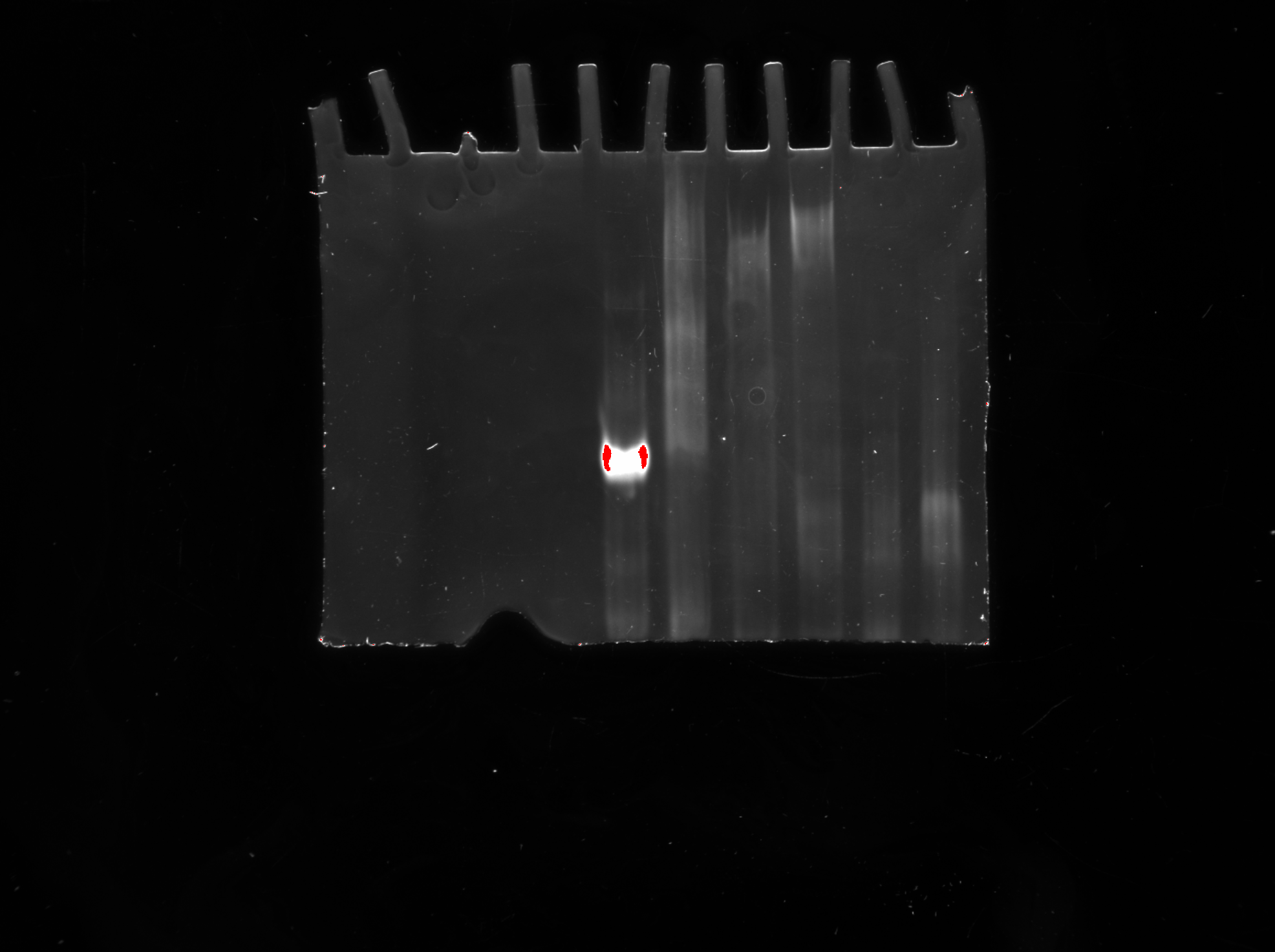

Supplement: Figure 4—figure supplement 1—source data 1. [file elife-70444-fig4-figsupp1-data1.zip › Figure 4-supplement 1-source data 1/111120_MatPmutants_matSvnsDNA_ethidium.tif]
